# A Survey of Prescription Errors in Paediatric Outpatients in Multi-Primary Care Settings: The Implementation of an Electronic Pre-Prescription System

**DOI:** 10.3389/fped.2022.880928

**Published:** 2022-06-09

**Authors:** Lu Tan, Wenying Chen, Binghong He, Jiangwei Zhu, Xiaolin Cen, Huancun Feng

**Affiliations:** Department of Pharmacy, The Third Affiliated Hospital of Southern Medical University, Guangzhou, China

**Keywords:** prescription errors, paediatric outpatients, primary care settings, electronic pre-prescription system, medication errors

## Abstract

**Background:**

Prescription errors impact the safety and efficacy of therapy and are considered to have a higher impact on paediatric populations. Nevertheless, information in paediatrics is still lacking, particularly in primary care settings. There exists a need to investigate the prevalence and characteristics of prescription errors in paediatric outpatients to prevent such errors during the prescription stage.

**Methods:**

A cross-sectional study to evaluate paediatric prescription errors in multi-primary care settings was conducted between August 2019 and July 2021. Prescriptions documented within the electronic pre-prescription system were automatically reviewed by the system and then, potentially inappropriate prescriptions would be reconciled by remote pharmacists *via* a regional pharmacy information exchange network. The demographics of paediatric patients, prescription details, and types/rates of errors were assessed and used to identify associated factors for prescription using logistic regression.

**Results:**

A total of 39,754 outpatient paediatric prescriptions in 13 community health care centres were reviewed, among which 1,724 prescriptions (4.3%) were enrolled in the study as they met the inclusion criteria. Dose errors were the most prevalent (27%), with the predominance of underdosing (69%). They were followed by errors in selection without specified indications (24.5%), incompatibility (12.4%), and frequency errors (9.9%). Among critical errors were drug duplication (8.7%), contraindication (.9%), and drug interaction (.8%) that directly affect the drug's safety and efficacy. Notably, error rates were highest in medications for respiratory system drugs (50.5%), antibiotics (27.3%), and Chinese traditional medicine (12.3%). Results of logistic regression revealed that specific drug classification (antitussives, expectorants and mucolytic agents, anti-infective agents), patient age (<6 years), and prescriber specialty (paediatrics) related positively to errors.

**Conclusion:**

Our study provides the prevalence and characteristics of prescription errors of paediatric outpatients in community settings based on an electronic pre-prescription system. Errors in dose calculations and medications commonly prescribed in primary care settings, such as respiratory system drugs, antibiotics, and Chinese traditional medicine, are certainly to be aware of. These results highlight an essential requirement to update the rules of prescriptions in the pre-prescription system to facilitate the delivery of excellent therapeutic outcomes.

## Introduction

Medication errors (MEs) are defined by the National Coordination Council for Medication Error Reporting and Prevention as any preventable incident that may cause or trigger inappropriate medication use or patient harm (1). The selection and administration of medication are under the control of health care professionals, patients, or consumers. However, medication errors can still be committed. As the most common MEs, prescription errors are considered a preventable source of iatrogenic diseases (2), and they could cause adverse consequences, including unnecessary conduct of diagnostic tests, prolonged hospitalisation time, inappropriate medical treatments, and even death (3).

Evidence reveals that paediatric subjects are particularly vulnerable because they are three times more likely than adults to have a potentially dangerous prescription error (4). Paediatrics who present unique demands to medication ordering are confronted with certain additional challenges. Dosage calculations in paediatrics are more prone to misjudgement because of the constant need for weight-based and body surface area-based dosing and unit conversion to small doses required for children. This situation is less common in prescribing medications to adults (5). Most drug dosages are primarily available for adults, and off-label ones are widespread and unlicensed, thus increasing the risk of preventable harm associated with medications (6). Moreover, various pharmacological factors, such as age-based variability in absorption, distribution, metabolism, and excretion of drugs, pose physiological vulnerabilities to the risk of overdosing or drug-drug interactions among children compared with adults (7).

Community-oriented primary care is an essential component of the healthcare system. In primary health care clinics, inappropriate prescription rates for paediatric populations vary from 13.5% to 70% (8, 9), and safety issues would compound without timely intervention to correct the problems. Knowing where and when errors are most likely to occur is generally assumed to be the first step to preventing these errors. Various studies have reported paediatric prescription errors among inpatients in tertiary care hospitals (10). However, the prevalence and characteristics of prescription errors in paediatric and neonatal outpatients in community-oriented primary care clinics are substantially lacking (8, 11). Therefore, it is essential to investigate the prescription errors in paediatric outpatients in community settings.

In contrast to hospital settings, community-oriented primary care centres suffer from a shortage of pharmacists who have professional experience in detecting paediatric prescription errors (i.e., drug interaction, drug allergy, and contraindication of certain medications). Herein, nurses sometimes play the role of pharmacy technicians in these settings (12, 13). Much effort has been devoted to addressing the shortage of professionals in primary care settings (14, 15). Liu et al. (16) developed a cloud-based pre-prescription review system with computer-assisted decision support and remote pharmacy for preventing prescription errors in community settings. Implementation of such an ancillary system can actively engage remote pharmacists in collaborating with primary care physicians in identifying and implementing preventative medication strategies. Thus, such a system may serve as an important aspect of rational drug therapy (17). The application of this system is still in its early stage. Moreover, the efficiency and accuracy of the preventative strategies of this system must be improved, especially for clinically managing paediatric outpatients.

Considering the vital role of prescription practises concerning paediatric outpatients, this study was conducted to determine the prevalence and characteristics of prescription errors based on a cloud-based pre-prescription review system across 13 community-oriented primary care settings in a certain region. In addition, this study aimed to identify the inappropriate medicines frequently prescribed to paediatric outpatients that will prevent errors during the prescription stage and serve as a basis for modifying the review rules of the system.

## Methods

### Study Design and Population

The cross-sectional study was carried out in thirteen community settings in Guangzhou from 1 August 2019 to 31 July 2021. All the medication orders were collected through the electronic pre-prescription system in the Department of Paediatrics, Internal Medicine, General Medicine, Ophthalmology, and Traditional Chinese medicine. The electronic transmission of prescriptions from primary-care physicians was examined automatically by a cloud-based pre-prescription system and potentially inappropriate prescriptions were transmitted to remote pharmacy staff in tertiary hospitals. Inappropriate medication orders should be corrected by the primary-care physicians before the dispensation. This study was approved by the ethics committee in our hospital.

### Inclusion and Exclusion Criteria

Inclusion criteria were set as follows. All prescriptions were prescribed in outpatient situations, and those of them without prescription errors were exactly excluded. The prescriptions should be medication-related (not laboratory-test orders), and the age of the participants should be lower than 18 years. Only a single medication order was prescribed for each subject during the study period. Self-prescription, inpatient, or adult was precluded. Prescriptions were not included in this study in case of the absence of confident medical data.

### Pre-Prescription Review Process

Pre-prescription review workflow was reported previously (16). When a medication order was received from a community setting, a real-time first review would be executed by the pre-prescription review system equipped with a rational commercial drug-use review program. Appropriate medication orders were admitted to printing, payment, and distribution. Reversely, two different conditions would come out if a potentially inappropriate prescription is found by the system. On the one hand, some inappropriate prescriptions confirmed by the system were immediately discontinued and fed back to the primary-care physicians with alert notes on the pop-up dialogue. Subsequently, the clinical physicians were required to make corrections and submit a renewed prescription. The altered medication orders needed to be reviewed once more.

For other potentially inappropriate prescriptions, on the other hand, which were not mechanically recognised by the electronic pre-prescription system, suspension and a further review by a remote pharmacist in tertiary hospitals would be required. In those cases, both prescription and patient information would be electronically sent to any pharmacy staff who were remotely available. The pharmacists should provide timely feedback within a flexible pre-set period (defined as 20 s in our application). Feedback from the clinical pharmacists on the interrupted medication orders was transmitted back to the primary-care physicians through a pop-up message. The prescribers could either revise the medication orders or rewrite the comments with an open-ended textbox.

### Assessment of Prescribing Appropriateness

Different scales of errors in the prescription of medications were defined and classified as errors of commission, omission, and other errors as delineated in previous reports (16, 18). The clinical drug use information was based on the drug instructions, clinical guidelines published both at home and abroad, and professionally recognised books. In this study, eleven primary description for prescription errors were categorised: indication for drug treatment not noticed, information missing, contraindication, inappropriate drug combination (the cause of prescription errors related to the selection of drugs with opposing pharmacological effects), non-conformity of the drug selection, duplicate therapy, dose selection (overdose or sub-therapeutic dose), inappropriate dosing frequency, inappropriate treatment duration, inappropriate drug formulation, and drug-drug interaction as shown in Table 1. Classifications of prescription errors were completed independently by two clinical pharmacists and checked by another pharmacist when they met the definitions.

**Table 1:** Classification and rates of prescription errors in this study.

**Types**	**Number(%)**	**Descriptions (or examples)**
**Errors of omission**		
Indication for drug treatment not noticed	709(24.5%)	Drugs prescribed without a specified indication.
Information missing	49(1.7%)	The clinical characteristics of patients (such as weight, age) and/or the disease information are unavailable.
**Errors of commission**		
Contraindication	26(0.9%)	Contraindications are specific medical reasons for not using a particular treatment for a medical condition in the usual way, such as allergy.
Inappropriate drug combination	24(0.8%)	Drugs prescribed with opposing pharmacological effects.
Non-conformity of the drug selection	357(12.4%)	Another drug is a preferred one because of it produces a superior clinical outcome, or reduces treatment cost or shows a lower risk.
Duplicate therapy	252(8.7%)	Two or more drugs prescribed concomitantly to achieve an additive pharmacological effect.
Overdose or sub–therapeutic dose	781(27.0%)	The dosage prescribed too high or too low for paediatric patients.
Inappropriate dosing frequency	286(9.9%)	The drug administration prescribed shorter interval times.
Inappropriate treatment duration	1(0.03%)	For example, more than 30 days chronic disease or in a prescription;
Inappropriate drug formulation	282(9.8%)	For example, patient received a oral drug that was packaged as by venous administration.
**Others**		
Drug–drug interaction alert	123(4.3%)	Concurrent use drugs may result in additive adverse reactions because of pharmacodynamic or pharmacokinetic interactions.

### Data Collection

Data were collected from the included prescriptions to be examined and characterised. The first step was the selection and categorisation step, in which all medication orders were screened to confirm their inclusion and exclusion criteria. After extracting the paediatric prescription, the prescriptions were classified based on the categorizations (Table 1). After that, the second step was to gather personal information and prescription information from each visit on a separate sheet.

The data-collection sheet was divided into two main parts. The first contained the individual items that should be available in each prescription (age, gender, weight, and diagnosis of the paediatric subjects) to enable the healthcare professionals to ensure whether the dose regimen was rational.

The second part of this section was gathering medication information from the collection sheet that contained several items on behalf of the various types of prescription errors. There may be more than one type of medication error found in a single prescription.

### Statistical Analysis

Data analysis was performed using Statistical Package for Social Sciences (SPSS version 20, IBM SPSS Statistic, Armonk, NY) software. The graphs were prepared using GraphPad Prism 8 (GraphPad Software Incorporated, San Diego, CA, USA). Continuous data were expressed as the mean ± standard deviation (SD), and categorical data were shown as frequency or percentage. The logistic regression model with the stepwise method was utilised to explore the potential relationship of independent variables with prescription errors. The dependent variable was defined as a paediatric outpatient visit that resulted in at least one prescription error. The independent variables included patient demographics (gender, age, weight), patient diagnosis, prescriber characteristics, and prescription details (prescription and medication characteristics; Tables 2, 3). Results were analysed using a *p* value (*p* < 0.05) as threshold for statistical significance.

**Table 2:** Characteristics of paediatric outpatients in this study.

**Characteristics**	**Number (%) or** **mean ±SD (range)**
**Patient characteristics**	
Gender (Male/Female)	968(56.1%)/756(43.9%)
Age (year)	6.3 ± 3.2 (1–18)
1–5	888(51.5%)
6–10	652(37.8%)
11–15	157(9.1%)
≥16	27(1.6%)
Weight (kg)	21.3 ± 10.8 (3.7–70.0)
Weight of patients aged 1–5	14.8 ± 3.8 (3.7–35)
Weight of patients aged 6–10	24.8 ± 7.4(7.0–60.0)
Weight of patients aged 11–15	38.6 ± 11.7(10.0–70.0)
Weight of patients aged ≥16	50.0 ± 11.3(37.0–67.0)
**Prescribers' specialty (top 5)**	
General practises	1226(71.1%)
Paediatrics	170(9.9%)
Internal medicine	126(7.3%)
Ophthalmology	76(4.4%)
Traditional Chinese medicine	65(3.8%)

**Table 3:** The characteristics of included prescriptions and the drug items in this study.

**Characteristics**	**Number (%) or** **mean ±SD (range)**
**Prescription characteristics**
Number of medications prescribed	3.5 ± 1.1 (1.0–6.0)
1	94(5.5%)
2	164(9.5%)
3	650(37.7%)
4	485(28.1%)
≥5	331(19.2%)
Diagnose (top 5)	
Acute upper respiratory infections of multiple or unspecified sites	980(47.6%)
Acute nasopharyngitis [common cold]	373(18.1%)
Acute tonsillitis	225(10.9%)
Acute bronchitis	154(7.5%)
Acute sinusitis, unspecified	85(4.9%)
**Medication characteristic**
Drug classification (top 5)	
Antitussives, expectorants, and mucolytic agents	1558(28.3%)
Cough and cold agents	938(17.1%)
Corticosterioids	858(15.6%)
Anti–infectives agents	715(13.0%)
Antiallergic agents	592(10.8%)
Gastrointestinal agents	154(2.8%)
Drug (top 5)	
Budesonide	624(11.3%)
Cefixime	460(8.3%)
Ipratropium bromide	410(7.5%)
Montelukast	305(5.5%)
Terbutaline	282(5.3%)

## Results

During the study period of 1 August 2019 to 31 July 2021, a total of 39,754 paediatric prescriptions were placed and screened *via* the electronic pre-prescription system. The study results investigated only the prescription-related errors in paediatric outpatients. In the current study, 38,030 out of 39,754 paediatric prescriptions were not included in this study because of prescriptions without errors (53.8%), repetitive medication orders for a single patient (18.4%), laboratory-test orders (12.6%), the absence of confident medical data (10.0%), and the inpatient situation (5.2%). Finally, 1,724 prescription errors met the inclusion and exclusion criteria of this study, representing 4.3%. The demographic characteristics (including gender, age, and weight) of paediatric patients and items for prescriptions were shown in Tables 2, 3.

**Figure 1:**
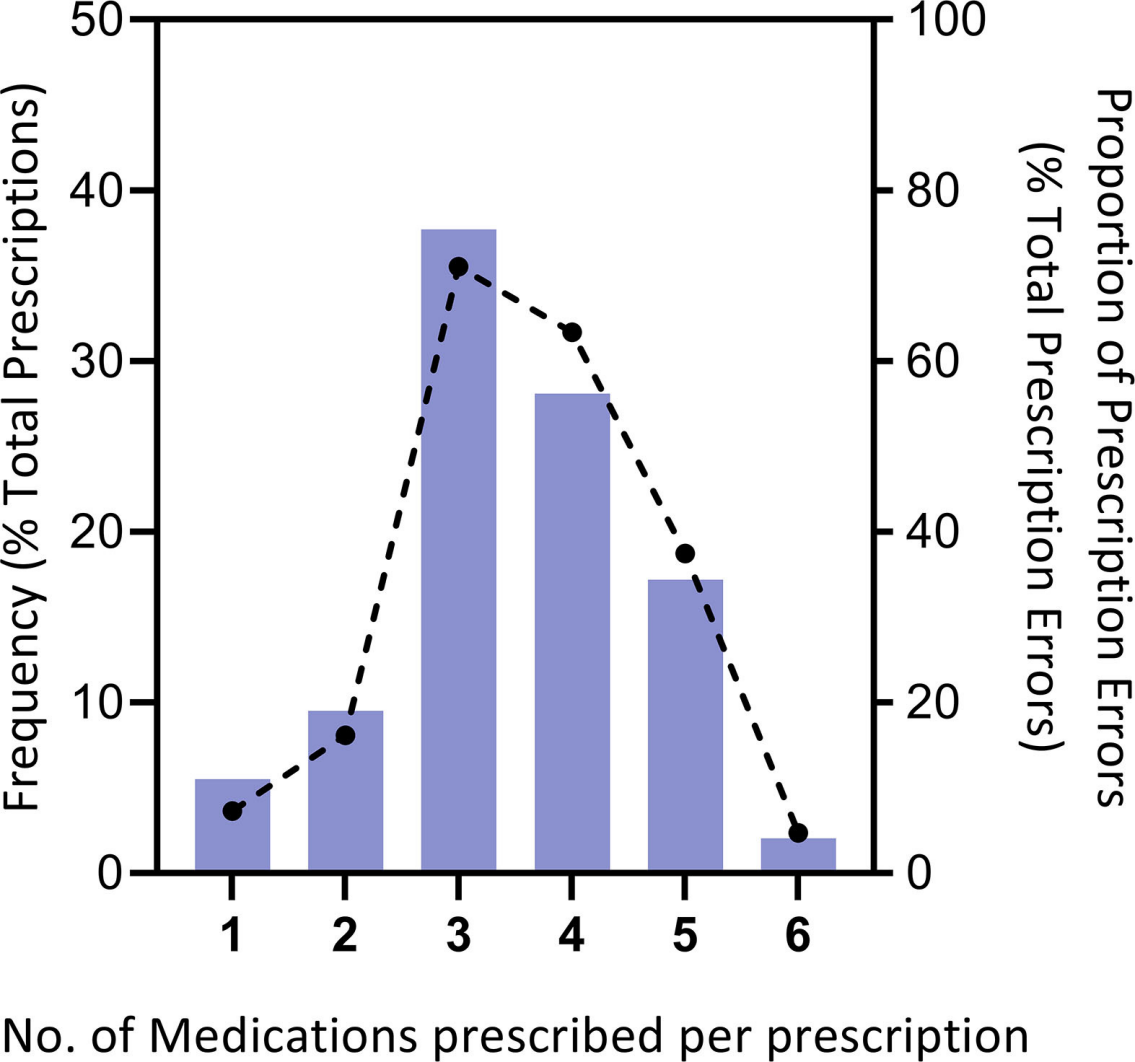
Distribution of prescriptions and errors per primary-care visit. The number of medications prescribed per patient visit (*n* = 1,724 patient visits)is approximately the proportion of prescription errors divided by the total prescription errors (total = 2890 errors).

Prescriptions from 1,724 paediatric patients (mean (SD) age 6.3(3.2), 968 males and 756 females) were analysed. The mean numbers of body-weight for participants were 21.3 ± 10.8 kg (ranging from 3.7 kg to 70.0 kg). Medication orders prescribed per patient visit were typically three drugs (37.7%). Prescriptions for 5 and 6 drug items were observed in 331 visits (17.2% and 2.0%, shown in Figure 1), and the proportion of prescription errors ranked second and third (18.7% and 2.35%, shown in Figure 1). Acute upper respiratory infections were the leading causes of treatment as they placed the largest numbers of prescriptions (980, accounting for 47.6%), followed by acute nasopharyngitis (373, accounting for 18.1%), acute tonsillitis (225, accounting for 10.9%), acute bronchitis (154, accounting for 7.5%), and acute sinusitis (85, accounting for 4.9%). Treatment medications were primarily classified as antitussives, expectorants, mucolytic agents (28.3%), and cough/cold agents (17.1%) which is consistent with the fact that the top five diagnoses were bronchus-associated diseases (Table 3).

In this cross-sectional survey, a total of 1,724 valid prescriptions (containing 6,002 drugs) were prescribed by 172 primary-care clinical practitioners, and all of them were examined by the pre-prescription system and clinical pharmacists. In these, 2,890 prescription errors were found. An average of 1.68 errors per medication order (range: 1–8) were observed. Regarding the types of error, dose errors (27.0%) ranked first in the incidence of prescription errors, followed by no indication for drug treatment (24.5%), non-conformity of the drug selection (12.4%), inappropriate dosing frequency (9.9%), and inappropriate drug formulation (9.8%) (shown in Table 1). Further analysis shows that sub-dose was served as the main type of dose error with an incidence of 69.0%.

**Figure 2:**
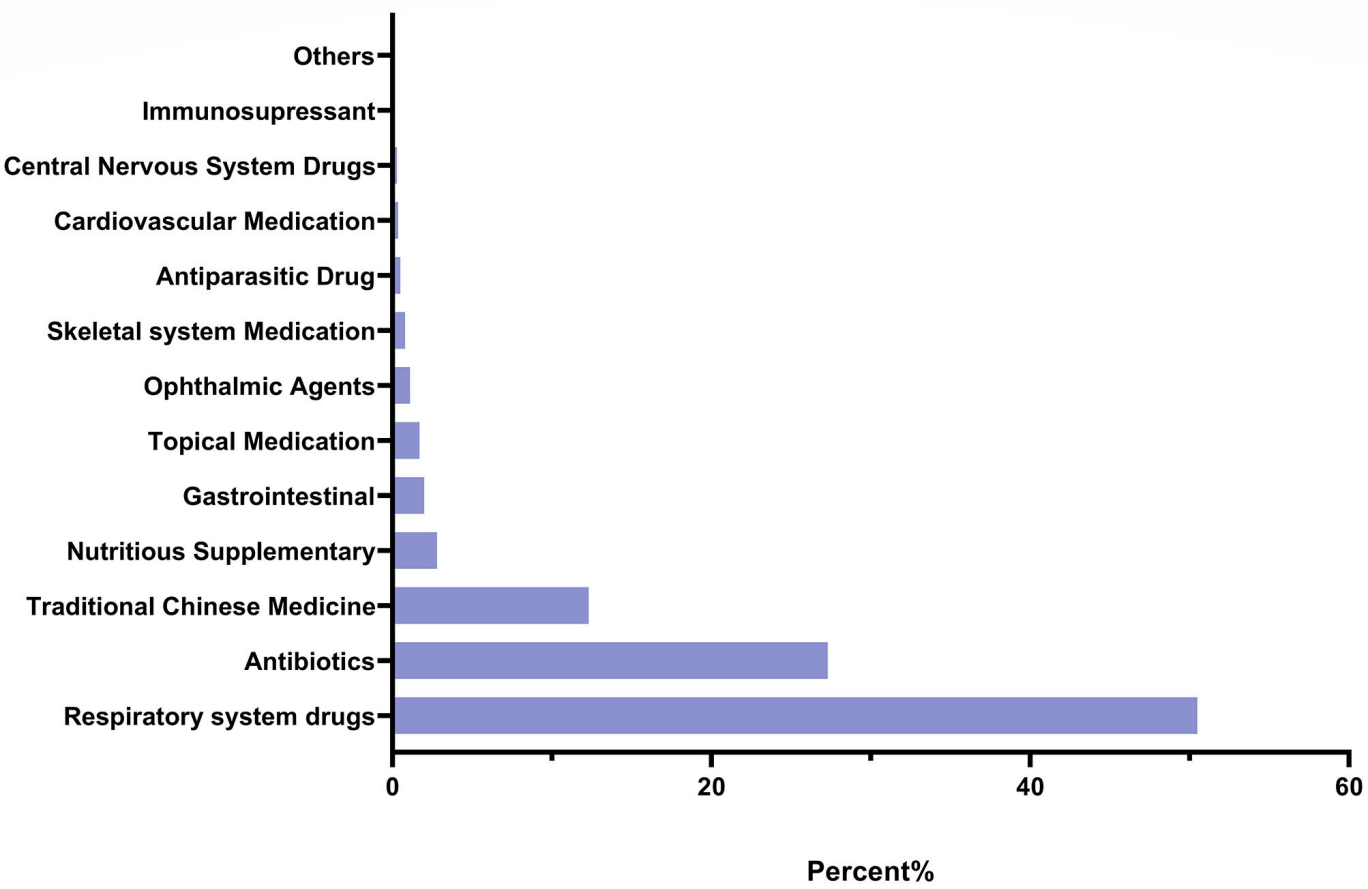
Incidence (%) of prescription errors under the different medication classifications.

In addition, it was found that the largest number of errors were associated with prescribing respiratory system drugs (50.5%), followed by antibiotics (27.3%), traditional Chinese medicines (12.3%), nutrition supplements (2.8%), gastrointestinal medications (2.0 %), topical medication (1.7%), ophthalmic agents (1.1%), skeletal system medications (0.8%), antiparasitic drugs (0.5%), cardiac medications (0.35 %), central nervous system drugs (0.29%), immunosupressants (0.12 %), and others (0.17%) (data shown in Figure 2).

**Figure 3:**
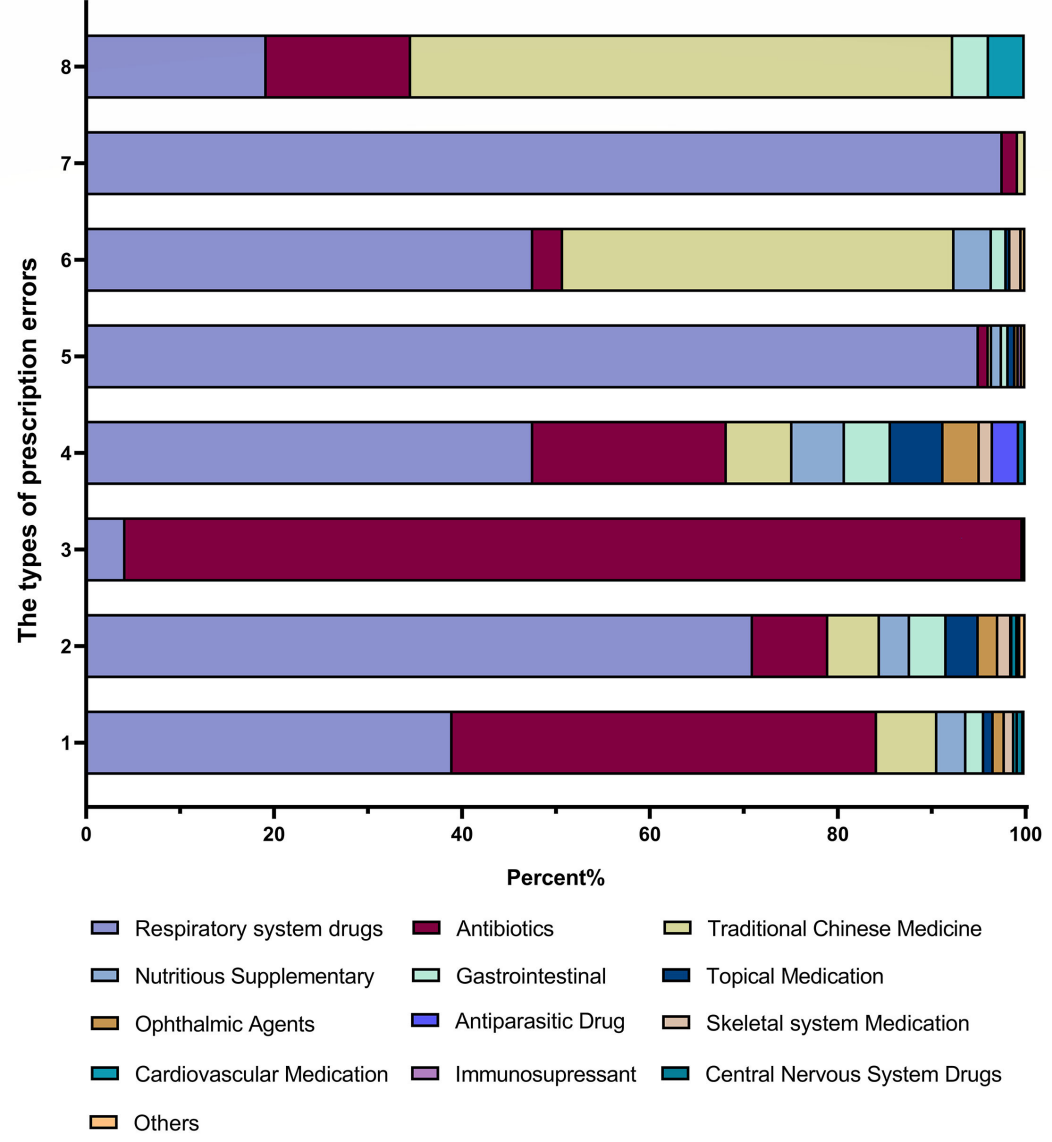
Types of prescription errors by medication classifications.

The class of medication was also categorised in each type of error. Figure 3 presented that the majority of drug dose errors (27.0%) occurred in prescribing antibiotics (45.2%), and such class of medication was also committed mainly in drug selection errors (95.5%). Indications for drug treatment not noticed were committed prescribing respiratory system drugs (71.0%), antibiotics (8.0%), traditional Chinese medicine (5.5%), nutrition supplements (3.2%) and gastrointestinal medications (3.9%). Inappropriate dosing frequency was observed in the prescription of respiratory system drugs (47.6%), antibiotics (20.6%), traditional Chinese medicine (7.0%), nutrition supplements (5.6%), and gastrointestinal medications (4.9%). Contraindication constituted more than half of the prescription of traditional Chinese medicine (57.7%), followed by respiratory system drugs (19.2%) and antibiotics (15.4%).

Independent variables associated with prescription errors were further explored by the logistic regression analysis with a stepwise method. Specific drug classification (antitussives, expectorants, and mucolytic agents, anti-infectives agents; *P* = 0.0019, OR = 1.412), patient age <6 years (*P* = 0.0021, OR = 1.785) and prescriber specialty (paediatrics; *P* = 0.036, OR = 2.113) related positively to errors. The following subgroup analysis of medication orders illustrated that prescriptions for antitussives (containing Chinese traditional medicine) and patient age <6 years were the only determinants positively related to prescription errors. Overdosing was the most frequently observed error in this subgroup analysis.

## Discussion

Medication errors related to outpatient paediatric prescriptions influence the efficacy or safety of the treatment, such as incorrect dosing (overdosing or under-dosing), contraindications, drug interactions, and absence of critical information. Prescription errors occur frequently in paediatric primary care settings, affecting patient safety and healthcare quality (8, 9). To the best of our knowledge, this study is the first to report paediatric prescription errors in community-oriented care services in our city. This cross-sectional study provides the characteristics and prevalence of paediatric subjects by analysing 39,754 outpatient paediatric prescriptions in community settings based on an electronic pre-prescription system. Of the 39,754 outpatient paediatric prescriptions, 1,724 prescription errors (4.66%) were identified. The majority of these errors were classified as dosing errors (27.0%), drug selection without specified indications (24.5%), incompatibility (12.4%), and frequency errors (9.9%). Therefore, the results of our study revealed that inappropriate medications (respiratory system drugs, antibiotics, and traditional Chinese medicines) frequently prescribed to paediatric outpatients should be considerably reduced with preventive measures.

Previously, the high frequency of prescription errors was attributed to the fact that, in primary-care settings, the medication orders could be limited due to a lack of professional resources and an electronic system for a medication review. With the pilot implementation of the pre-prescription review system in community settings (14, 16, 19), the occurrence and frequency of medication errors were reduced in other areas, highlighting that remote pharmacy services directly improved the medication process. The main advantage of applying the pre-prescription system was the prevention of drug misuse which could result in serious health issues (20). In the present study, after the pilot implementation of the pre-prescription review system at 13 primary care settings, the paediatric prescriptions for outpatients could be mandatorily reviewed by the system, and the potentially inappropriate prescriptions were transmitted to remote pharmacists in tertiary hospitals. Advanced review software and customisation can help timely intervention for inappropriate prescription errors before printing and payment, as well as overcome the shortage of healthcare professionals in community care settings. In addition, the implementation of a pre-prescription system can, to some extent, reduce the workload of pharmacists and improve the efficiency of prescription reviews. A total of paediatric prescriptions (*n* = 39,754) were collected during the study period. In these, 1,724 inappropriate prescriptions (1,724/39,754, 4.3%) were found, and the rate of prescription errors may be lower than the previously reported error rate (8, 9). A large variation was found in the prescribing error rates as reported previously. The results are due to the different definitions for prescription errors and different methods to calculate the incidence rates.

The first part of prescription errors was classified as missed essential demographic data and medical status of paediatric subjects, which included all types of omission such as patient's weight, age, and patients diagnosis from paediatric prescriptions. However, none of the demographic data was missing in the current study. The missed diagnosis is also essential data for medication but in this study, only 49 of 39,754 prescriptions were found, which was inconsistent with the finding of other studies. The prescriptions lacking patient diagnosis should be returned to the primary-care physicians with alerting notes by the pre-prescription system. Cooperation between electronic systems and clinical pharmacists is effective in reducing prescription errors, analysing reported errors, and facilitating interventions to improve therapeutic outcomes in an outpatient paediatric practise.

The substantial proportion of incorrect dosing (overdosing or under-dosing, 27.0%) in patients raises great concern, which was in line with the findings of most previous studies (21–23). Paediatrics pose a unique set of risks of dosing errors in paediatric general wards, predominantly because of the frequent need for dose calculations required based on subjects' weight, body surface area, and age. Weight-based dosing is possibly a crucial factor contributing to the rate of dosing errors (24). For example, healthcare professionals have difficulties in making calculations that could contribute to incorrect dosing (25). Subsequently, in our further analysis, dosing errors with underdoses generally outnumbered those with overdoses, which was inconsistent with the results across different settings and types (26–28). A potential reason for underdose errors is that some prescribers may have decreased the dose for paediatrics owing to their empiric dosage reduction. Empiric dosage reduction is inappropriate since multiple pharmacodynamic and pharmacokinetic studies have illustrated that a high-dose schedule is essential to sufficiently treat disease (29).

Furthermore, the absence of valid indications for drugs in prescription (24.5%) for patients was the second error in the current study, while it was recognised as the most common inappropriate drug prescription in Soraya's study (30). Perhaps because the previous study had been conducted in a sample of nursing home residents where the non-pharmacist workers such as nurses played the role of pharmacy technicians and operated primarily as dispensers.

There were additional noteworthy observations found in our data analysis. The number of prescribed medications ranged from 1 to 6 drug items per visit, but with an average of 3.5 ± 1.1 drugs per medication order. An increased number of prescribed drugs is related to increased medication discrepancies at dispense, underlining the need to treat polypharmacy as a multifaceted risk to patient health (31). This was obvious from the results that reflected a difference in medication errors between orders that included a large number of drugs and those with low numbers. The rates of medication error were more likely to occur higher in prescriptions with three, four, and five drug items (Figure 1). While our study design did not allow us to confirm the reasons for this, it is possibly supported by empirical evidence from primary-care physicians.

Another finding was the predominance of respiratory system drugs (antitussives, expectorants, and mucolytic agents) were prescribed in paediatric patients, which are in accord with the findings in the literature (18). It could be explained by their wide use in acute upper respiratory infections which commonly occurred in paediatric patients in community settings. Antibiotics were the fourth most commonly prescribed medication group in our study, but it was often involved in prescription errors (27.3%). Nevertheless, error rates in other studies were often higher than 15% (32). With the regard to the third commonly prescribed medication, traditional Chinese medicines showed that prescription errors accounted for 12.3% of all errors. The evaluation of traditional Chinese medicines has not been revealed in any study before.

## Conclusions

In conclusion, evidently, our study provided the prevalence and characteristics of prescription errors of paediatric outpatients in community settings based on an electronic pre-prescription system. A considerable number of wrong dose errors (27.0%) propagated throughout the pre-prescription system, while 69.0% of under-dose errors occurred during treatment. Dose calculation for paediatrics in drug therapy is certainly to be aware of, especially the underdose errors. Awareness of inappropriate medications (respiratory system drugs, antibiotics, and traditional Chinese medicines) frequently prescribed to paediatric outpatients should prevent errors during the prescription stage.

## Data Availability Statement

The raw data supporting the conclusions of this article will be made available by the authors, without undue reservation.

## Author Contributions

LT and WC wrote the manuscript. LT, BH, and HF designed the research. LT, WC, BH, XC, and HF performed the research. LT and WC analysed the data. All authors have given their final approval for the manuscript.

## Funding

This study was supported by the Medical Scientific Research Foundation of Guangdong Province, China (No. C2021059).

## Conflict of Interest

The authors declare that the research was conducted in the absence of any commercial or financial relationships that could be construed as a potential conflict of interest.

## Publisher's Note

All claims expressed in this article are solely those of the authors and do not necessarily represent those of their affiliated organizations, or those of the publisher, the editors and the reviewers. Any product that may be evaluated in this article, or claim that may be made by its manufacturer, is not guaranteed or endorsed by the publisher.
